# Alarming Rise of Obesity: The 4^th^ United Nations High-Level Meeting on Noncommunicable Diseases and Mental Health Should Advance Action to Tackle Obesity

**DOI:** 10.5334/gh.1459

**Published:** 2025-08-28

**Authors:** Shanthi Mendis, Ian Graham, Francesco Branca, Tea Collins, Collin Tukuitonga, Asela Gunawardane, Jagat Narula

**Affiliations:** 1 The Geneva Learning Foundation, Geneva, Switzerland; 2 Cardiovascular Medicine, Trinity College, Dublin, Ireland; 3 University of Geneva, Geneva, Switzerland; 4 World Health Organization, Geneva, Switzerland; 5 The University of Auckland Center for Pacific and Global Health, New Zealand; 6 Ministry of Health and Indigenous Medicine, Colombo, Sri Lanka; 7 University of Texas Health Sciences Center, Houston, Texas, USA

**Keywords:** Obesity, overweight, prevention, cardiovascular disease, noncommunicable diseases, physical activity, unhealthy diet, United Nation High Level Meeting, World Health Organization, accountability, sustainability

## Abstract

Obesity is a growing global crisis increasing the risk and outcomes of a range of noncommunicable diseases including cardiovascular diseases, type 2 diabetes, cancer, chronic respiratory disease, steatotic liver disease, and kidney disease.

Obesity in children tracks into adulthood increasing their risk of noncommunicable diseases including cardiovascular diseases.

A growing body of evidence confirms that there are affordable and scalable policies to promote a healthy diet and regular physical activity to prevent overweight and obesity including in children and adolescents.

Despite the burden caused by obesity and its preventability, the topic does not appear to be a priority on the agenda of the global public health community and implementation of public health policies to prevent obesity at country level has been patchy.

At the upcoming United Nations 4^th^ High Level Meeting on noncommunicable diseases, Heads of State and Government need to go beyond making political commitments to prevent obesity and, take concrete steps to increase and monitor budget allocations for implementing policies for population wide prevention of physical inactivity and unhealthy diet.

Obesity is a chronic disease that affects over one billion people in the world and is a major risk factor for cardiovascular disease, type 2 diabetes, and cancer. It is impossible to advance prevention and control of noncommunicable diseases, without simultaneously halting the rise of obesity. The 2018 political declaration of the 3^rd^ United Nations General Assembly High-Level Meeting on noncommunicable diseases calls for the Implementation of cost-effective and evidence-based interventions to halt the rise of overweight and obesity, especially childhood obesity. Since then, the evidence supporting the impact of regular physical activity and a healthy diet on the prevention of obesity has become more compelling. However, the prevalence of obesity across all age groups has increased due to the ineffective public policy response, the fierce opposition from commercial actors, and difficulties in navigating implementation challenges. This paper outlines the growing evidence, recent developments, and lessons learnt since 2018 and highlights new opportunities and remaining challenges with regard to prevention of obesity, ahead of the 4th United Nations High-Level Meeting on noncommunicable diseases in September 2025.

## Introduction

Obesity is a chronic disease. Although obesity is largely preventable, its prevalence has increased in all age groups during the last two decades. In 2022, over one billion people aged 5 years and over were living with obesity worldwide (1). Sixteen percent of adults (18 years and older) and 8% of children and adolescents (aged 5–19 years) were affected by obesity. Among children under the age of 5 years, 5.6% (estimated 35 million) were affected by overweight or obesity. From 1990 to 2022, the percentage of children and adolescents living with obesity increased four-fold globally, while the percentage of adults living with obesity more than doubled (1,2). The disease burden due to overweight and obesity also increased over this period at an annual rate of 0.48%, reaching 128 million DALYs in 2021 (3). While the prevalence of obesity has been growing in Low-and Middle-Income Countries (LMIC), undernutrition has not been fully addressed, leading to a double burden of malnutrition (4).

Obesity increases the risk of a range of Noncommunicable Diseases (NCDs) including cardiovascular diseases, type 2 diabetes, cancer, chronic respiratory disease, steatotic liver disease, and kidney disease (5,6,7,8) (Table 1). Obesity in children tracks into adulthood, increasing their risk of various NCDs such as type-2 diabetes and cardiovascular diseases (5). Obesity also has an adverse impact on blood pressure, blood sugar, lipids, cardiac structure, and function (5,6). In addition, obesity increases the risk of developing several cancers, including breast, colorectal, endometrial, kidney, oesophageal, pancreatic, liver, and gallbladder cancer (7). Further, living with obesity is associated with limitations of daily activities, such as walking or other basic activities of daily living, to impairment of mental wellbeing and reduction of occupational opportunities (1,2,8).

**Table 1:** Adverse health consequences of obesity (5,6,7,8).


CATEGORY	DISEASE/CONDITION

Cardiovascular	• Hypertension • Coronary artery disease • Stroke • Heart failure • Dyslipidemia

Metabolic and endocrine	• Type 2 diabetes mellitus • Insulin resistance • Metabolic syndrome • Polycystic ovary syndrome

Respiratory	• Obstructive sleep apnoea • Obesity hypoventilation syndrome • Asthma

Cancer	• Breast cancer • Endometrial cancer • Colon cancer

Gastrointestinal and liver	• Non-alcoholic fatty liver disease [Metabolic Dysfunction-Associated Steatotic Liver Disease (MASLD)] • Non-alcoholic steatohepatitis [Metabolic Dysfunction-Associated Steato Hepatitis (MASH)] • Gallstones • Gastroesophageal reflux disease

Musculoskeletal	• Osteoarthritis • • Lower back pain • Gout

Mental health	• Anxiety disorders • Depression • Eating disorders

Reproductive	• Infertility • Menstrual irregularities


Growing levels of overweight and obesity are contributing to the NCD burden and premature mortality due to NCDs (2,8). Modelling analyses conducted in middle -income countries with rising levels of obesity indicate that control of overweight and obesity has a strong potential to reduce the NCD burden (9,10). Currently, no country is on track to meet the global target to stop the rise of obesity (8). Rising obesity trends will in addition undermine the likelihood of countries attaining the SDG target 3.4 of reducing premature mortality from NCDs by one third by 2030 (2,4,8).

It is estimated that the total cost of overweight and obesity to health services globally is US$ 990 billion per year, over 13% of all healthcare expenditure (11). Obesity also results in indirect costs due to impaired productivity, disability, lost life years, and reduced quality of life (4,6,8).

## Causes of overweight and obesity

Causes of obesity have been summarised by the World Health Organization (WHO) (1,12,13).

Overweight and obesity result from an imbalance of energy intake (diet) and energy expenditure (physical activity). In most cases, obesity is a multifactorial disease due to obesogenic environments and psycho-social factors. In a minority of patients, single major aetiological factors can be identified (medications, diseases, immobilization, iatrogenic procedures, monogenic disease/genetic syndrome).

Obesogenic environments increase the likelihood of weight gain because of structural factors that limit the availability of healthy food at locally affordable prices, lack of safe and easy physical mobility for daily living, and absence of an adequate legal and regulatory environment. At the same time, the lack of an effective health system response to identify excess weight gain and fat deposition in their early stages is aggravating the progression to obesity.

## Obesity is preventable: growing body of evidence on physical activity and healthy diet

Regular physical activity and diets rich in whole grains, legumes, nuts, vegetables, and fruits lower the risk of developing obesity (14,15). In contrast, high intake of free sugars, particularly from sugar-sweetened beverages, is associated with an increased risk of overweight and obesity (16).

A growing body of evidence confirms that a healthy diet and regular physical activity prevent overweight and obesity including in children and adolescents (17,18,19). A systematic review of 172 studies assessed the effectiveness of obesity interventions targeting children aged 5–11 years. Physical activity interventions alone or combined with diet were found to have a positive impact on preventing excess weight gain (17). Another systematic review of 74 studies found that physical activity interventions prevent excess weight gain in children and adolescents aged 12–18 years (18). A recent meta-analysis of 73 randomised controlled studies analysed the effect of physical activity, nutrition, and psychological interventions in participants under 18 years. Physical activity, diet, and multicomponent interventions had beneficial impacts on body mass index, lipids blood pressure, and insulin resistance (19).

Evidence on the effectiveness of fiscal policy interventions on the prevalence and incidence of overweight and obesity has become compelling (20,21,22). A systematic review of 17 studies found that taxation increased the probability of purchasing healthy beverages (20). Another systematic review of 11 studies found that taxing foods with added sugar reduced their consumption (21). In 2021, WHO recommended taxation of sugar-sweetened beverages and implementation of fiscal and pricing policies to promote healthy diets (22). The WHO Global guidelines on physical activity provide recommendations for regular physical activity for children, adolescents, adults, and people living with chronic conditions and disabilities (14).

## Interventions and recommendations to address obesity

Treatment of obesity within the healthcare system is currently costly and resource-intensive. Modalities available for treatment of adult obesity at the individual level include clinical counselling focused on diet, physical activity, and behaviour change, pharmacotherapy, and bariatric surgery (23).

Several classes of medicines have been used for pharmacological treatment of obesity, including those that limit nutrient absorption (e.g., orlistat); insulin sensitizers and suppressors (e.g., metformin, octreotide, exenatide); anorectic agents (e.g., sibutramine, leptin, phentermine, benzphetamine); and other stimulants (e.g., thyroid hormone, dinitrophenol, amphetamine, fenfluramine, dexfenfluramine, phenylpropanolamine). These medicines have led to average weight loss of 3–9% relative to placebo (23).

More recently, a new class of medicines has been authorised for the management of obesity, the glucagon-like peptide-1 (GLP-1) receptor agonists (tirzepatide, semaglutide, liraglutide) (23). Evidence-based guidelines recommend their use only after dietary, exercise and behavioural approaches have been used and evaluated and always alongside a reduced-calorie diet and increased physical activity. Medicines are discontinued if after 3–6 months at least 5% of the initial body weight has not been lost. They are not recommended for children under the age of 12 years (23). This class of medicines can lead to 5–15% weight loss, but usually requires ongoing use to maintain weight loss and the safety of their long-term use needs to be ascertained (24). They may also not be affordable for the public health sector, leading to inequities in access. Stringent prescription restrictions may be needed to prevent misuse of anti-obesity medicines for cosmetic weight loss (25). Further, the concept of ‘chemical salvage’—using an expensive drug to combat a life-style related problem—is neither logical nor sustainable, especially for LMIC.

Although bariatric surgery can be effective for the treatment of severe obesity, there are medical, nutritional, surgical, and psychological risks and complications associated with the procedure (23). For most LMIC, offering bariatric surgery services will not be feasible in the public sector due to cost implications and a lack of specialist obesity management services.

Outcome targets have been endorsed for controlling obesity by the World Health Assembly in 2013 and the United Nations General Assembly in 2014 (26,27). The WHO Commission on Ending Childhood Obesity issued recommendations to address childhood obesity in 2016 (12). More recently, at the 75th World Health Assembly in 2022, Member States adopted new recommendations for the prevention and management of obesity and endorsed the WHO Acceleration Plan to Stop Obesity (13). However, implementation of these recommendations is lagging behind.

## Patchy country action and implementation challenges

The results of periodic NCD country capacity surveys conducted by WHO since 2001 demonstrate that Actions recommended by the WHO’s Global NCD Action Plan for addressing obesity are feasible (26,28). Implementation challenges identified in these surveys include inadequate adoption of fiscal policies, such as taxation on sugar-sweetened beverages and unhealthy foods and weak multisectoral mechanisms for policy coherence.

Exclusive breastfeeding in the first six months of life and continued breastfeeding lower the risk of overweight and obesity, in addition to other health benefits (12,13). To protect breastfeeding from commercial milk formula marketing, the International Code of Marketing of Breast-milk Substitutes was adopted in 1981, by the World Health Assembly. Currently, 144 countries have adopted the Code into national law, with 32 of them having laws that substantially align with the Code (29). Four decades following the Code adoption, aggressive commercial milk formula marketing, in violation of the Code, persists globally, even in countries with legal measures in place (30).

Policy interventions to increase the cost of sugar-sweetened beverages to the consumer or to reduce the sugar content of the drinks have been introduced in several countries, including Mexico, Saudi Arabia, South Africa, and the United Kingdom of Great Britain and Northern Ireland. They have been effective in either reducing sales or reducing consumption of sugar from sugary drinks (31,32,33,34). Other countries, such as Norway, Hungary, India, Denmark, Bermuda, Dominica, St. Vincent and the Grenadines, and the Navajo Nation (USA), have implemented taxes on unprocessed sugar and sugar-added foods to decrease their consumption (13). As of 2024, 15 Pacific Island Countries have introduced taxes on unhealthy food and beverage consumption (35).

The impact of regulations to restrict marketing foods to children have been evaluated in studies conducted in several countries (Australia, Canada, Chile, European Union, Germany, Mexico, Singapore, South Korea, Spain, United Kingdom, and United States) (36,37). Others, (Australia, Belgium, Canada, Denmark, France, Germany, Ireland, Israel, Italy, New Zealand, Norway, Spain, Sweden, South Korea, Switzerland, United Kingdom, and United States) have implemented and evaluated programs to promote healthy diet and physical activity in public institutions (38,39). These efforts are leading to encouraging developments in the fight against obesity. Policy implementation at country level shows that enforcement challenges, industry interference, and resource constraints have hindered full policy implementation for prevention of obesity, underscoring the need for robust regulatory frameworks, better monitoring, and sustained political commitment (12,13).

Environments that people live in often promote unhealthy diet and physical inactivity, because they are manipulated by pervasive marketing of products high in fats, sugars, and salt. In addition, healthy dietary choices are unaffordable to about one third of the world population (8,12,13). Screen viewing (i.e., watching TV, using computers, tablets, smartphones, and playing on game consoles) has become a desirable behaviour increasing sitting time (12). In addition, manufacturers of processed foods and the restaurant industry use widespread digital marketing and lobbying without any accountability (12,14). Further, tobacco companies have acquired leading food manufacturing companies and have developed food products that are engineered to stimulate the brain’s neural pathways related to pleasure and reinforcement (41).

Despite these disturbing developments, governments are disinclined to take regulatory action or implement policies to counteract the actions of the food industry that are harmful to public health. Further, civil society has not been successful in pressurising governments to take meaningful political action or increasing the public demand for obesity prevention policies (8,12,13,40). All these reinforce sedentary behaviour and preferences and demands for foods of poor nutritional quality. Political will, government leadership, and accountability of the food industry are critical for overcoming these challenges that continue to hamper the progress of the prevention of obesity (8,40).

In LMIC, the effect of setting policies to prevent obesity is often short-lived due to insufficient long-term funding and health workforce capacity for their implementation (27,42,43). The average health spending per capita in low-income, lower- middle-income, upper-middle-income, and high-income countries is approximately US$ 43, 132, 540, and 3,731 respectively (42). In 2021, around 47% of the global population lived in countries where health spending per capita was below US$ 200. Currently, investment in health remains significantly below the US$ 86 per capita benchmark recommended for achieving universal primary healthcare. Further, WHO estimates have projected a shortfall of 18 million health workers by 2030, mostly in LMIC (43).

## Sustainable and scalable solutions

Sustained political will, financial resources, and a skilled health workforce are essential to navigate the complexity of implementation challenges alluded to above and deliver results. As underscored in the WHO Acceleration plan to stop obesity (13), to make it easy for people to eat a healthy diet and be physically active a range of regulatory, fiscal and policy interventions need to be implemented together so that they are complementary.

Approaches endorsed in the WHO Acceleration plan to stop obesity (3) include:

- policies to promote breastfeeding and improve the early childhood food environment
- policies to protect people from the harmful impact of food marketing
- nutrition labelling policies (including front-of-pack labelling)
- fiscal policies (including taxes and subsidies to promote healthy diets)
- policies to establish healthy public food procurements
- policies to promote the reformulation of manufactured food
- policies to promote physical activity in schools and
- policies to include the diagnosis and treatment of obesity in the basic health care package

Several measures are necessary for successful adoption of these approaches. First, priority has to be given to interventions that are affordable and scalable, particularly in LMIC. For example, investment in population-wide obesity prevention (low cost with long-term and wide-ranging benefits) need to be much higher than expenditure incurred for obesity treatment (shorter-term gains but higher costs and potential adverse effects) (26,27). The implementation capacity and availability of financial and workforce resources need to be given due consideration in selecting policies. The global NCD action plan includes physical activity and healthy diet interventions among the 16 very cost-effective high impact interventions (cost-effectiveness ratio ≤I$100 per DALY averted) (“best buys”) (26,27). In addition, disease specific health accounts are critical for monitoring spending levels on prevention activities including the implementation of obesity prevention interventions. It is of note that a substantial majority of countries worldwide are currently not generating (or not reporting) these data (44).

Second, Government regulatory interventions should not be replaced by industry self-regulation as there is no evidence that relying on industry self-regulation alone will be effective (45,46). Although currently there are more industry-led pledges on food advertising to children than government regulations, there are major concerns regarding their enforcement (47,48).

Third, obesity prevention policies need to be embedded in National Multisectoral Action Plans and implemented at subnational levels engaging multiple sectors and stakeholders while managing conflicts of interest. It is critical to mobilize the support of communities for policy implementation so that simultaneous attention is directed at top-down as well as bottom-up drivers and supply, as well as demand forces (26,27).

Based on evidence supporting the Global Action Plan for NCDs, WHO has developed an obesity technical package to assist countries in their decision-making process (13,26). It helps to prioritize proven policy interventions using country-specific demographic data and impact estimates. Thirty-four frontrunner countries across the six WHO regions are receiving technical support from WHO until 2030 to generate evidence and expertise for future expansion of the acceleration plan to stop obesity.

## The Fourth United Nations High-Level Meeting on NCDs: an opportunity

Currently, the global health community is preparing for the 4^th^ High-Level Meeting of the United Nations General Assembly on prevention and control of NCDs, in September 2025. During the three United Nations high-level meetings, in 2011, 2014, and 2018 (27), heads of state and government have made 63 commitments to accelerate national action for prevention and control of NCDs including obesity. In 2018, the 3^rd^ UNHLM made a commitment to ‘*Implement cost-effective and evidence-based interventions to halt the rise of overweight and obesity, in particular childhood obesity, taking into account WHO recommendations and national priorities’* (27). However, these commitments have not been operationalized in a timely manner to meet time-bound SDG and NCD targets, including the target to halt the rise in obesity.

There is little hope of stopping the rise in obesity levels or tackling NCDs unless governments take the lead and strengthen accountability of all key stakeholders. There must be a clear expression of a political position challenging the manufacturers of ultra-processed foods, who have not demonstrated any serious commitment “to be part of the solution” (40,49). Concentration, financialization, and corporatisation of the food system are constantly increasing and shareholders block any attempts of CEOs to direct companies towards greater social responsibility (50).

## Conclusion

More than a decade ago, the world adopted a target to halt the rising trend of obesity. However, the prevalence of obesity and the number of affected individuals continue to rise in all age groups and in all countries at an alarming rate. Obesity tracks with age and is difficult and costly to reverse through pharmacological means. Overweight and obesity are undermining the progress of NCD prevention and control.

All countries need to embed policies recommended in the WHO’s *“accelerated plan to stop obesity”* in national multisectoral NCD plans, with context-specific adaptations (13). Regular physical activity and a healthy diet need to be prescribed to all age groups, as “*safe and affordable medicines”*, through population-wide policies. In addition to preventing obesity, physical activity and a healthy diet provide a wide range of health, social, and economic benefits and a high return on investment to countries at all levels of development (13,14,26,27). At the United Nations 4^th^ High-Level Meeting on NCDs, Heads of State and Government are urged to go beyond making political commitments. As a first step, considering the need to increase and monitor budget allocations for population wide prevention of physical inactivity and unhealthy diet is absolutely essential, if Heads of State and Government are serious about responding to the alarming rise in obesity as well as the insufficient progress in addressing NCDs.
